# Improve definition of titanium tandems in MR-guided high dose rate brachytherapy for cervical cancer using proton density weighted MRI

**DOI:** 10.1186/1748-717X-8-16

**Published:** 2013-01-17

**Authors:** Yanle Hu, Jacqueline Esthappan, Sasa Mutic, Susan Richardson, Hiram A Gay, Julie K Schwarz, Perry W Grigsby

**Affiliations:** 1 Department of Radiation Oncology, Washington University School of Medicine, Saint Louis, Missouri, USA

**Keywords:** Cervical cancer, MRI, HDR Brachytherapy, T2 weighting, Proton density weighting

## Abstract

**Background:**

For cervical cancer patients treated with MR-guided high dose rate brachytherapy, the accuracy of radiation delivery depends on accurate localization of both tumors and the applicator, e.g. tandem and ovoid. Standard T2-weighted (T2W) MRI has good tumor-tissue contrast. However, it suffers from poor uterus-tandem contrast, which makes the tandem delineation very challenging. In this study, we evaluated the possibility of using proton density weighted (PDW) MRI to improve the definition of titanium tandems.

**Methods:**

Both T2W and PDW MRI images were obtained from each cervical cancer patient. Imaging parameters were kept the same between the T2W and PDW sequences for each patient except the echo time (90 ms for T2W and 5.5 ms for PDW) and the slice thickness (0.5 cm for T2W and 0.25 cm for PDW). Uterus-tandem contrast was calculated by the equation C = (S_u_-S_t_)/S_u_, where S_u_ and S_t_ represented the average signal in the uterus and the tandem, respectively. The diameter of the tandem was measured 1.5 cm away from the tip of the tandem. The tandem was segmented by the histogram thresholding technique.

**Results:**

PDW MRI could significantly improve the uterus-tandem contrast compared to T2W MRI (0.42±0.24 for T2W MRI, 0.77±0.14 for PDW MRI, p=0.0002). The average difference between the measured and physical diameters of the tandem was reduced from 0.20±0.15 cm by using T2W MRI to 0.10±0.11 cm by using PDW MRI (p=0.0003). The tandem segmented from the PDW image looked more uniform and complete compared to that from the T2W image.

**Conclusions:**

Compared to the standard T2W MRI, PDW MRI has better uterus-tandem contrast. The information provided by PDW MRI is complementary to those provided by T2W MRI. Therefore, we recommend adding PDW MRI to the simulation protocol to assist tandem delineation process for cervical cancer patients.

## Background

Cervical cancer ranks the third in the United States based on the average number of life years lost due to cancer [1]. It continues to be a significant problem in the United States and worldwide. In our clinic, cervical cancer patients are treated with daily external beam radiation therapy and weekly high dose rate (HDR) brachytherapy [2-4]. In HDR brachytherapy, radiation is delivered through a “tandem and ovoid” applicator. Knowledge of precise locations of both the tumor and the applicator relative to internal anatomy is essential to ensure radiation delivery accuracy, which may turn into improved local control and reduced morbidity.

Imaging modalities used for treatment planning for cervical cancer patients include computed tomography (CT) [5,6] and magnetic resonance imaging (MRI) [7-12]. Currently, CT is considered as the standard imaging modality for radiotherapy treatment planning for cervical cancer. It is fast and has a good spatial resolution. However, CT has poor soft tissue contrast, which makes it very challenging to delineate the tumor from surrounding tissues. MRI has great soft tissue contrast. It offers a variety of sequences such as T2 weighted (T2W) MRI and diffusion weighted imaging (DWI) to enhance the contrast between tumors and surrounding tissues. With better tumor-tissue contrast, it is possible to customize the treatment for each fraction based on the size and location of the cervical tumor. In addition, MRI does not involve any ionizing radiation and won’t add radiation dose to patients. Those advantages make MRI highly desirable in radiation oncology, especially in pelvic area such as cervical cancer.

The “tandem and ovoid” applicator used to treat cervical cancer is made of titanium. It is light, strong and compatible with the MRI imaging modality. However, it does introduce artifact to MRI images. Titanium is paramagnetic whereas soft tissues are in general slightly diamagnetic. When titanium tandems are placed in cervical cancer patients treated with HDR brachytherapy, the susceptibility difference between the titanium tandems and surrounding soft tissues can result in local magnetic field gradients along the interface, causing signal loss and geometric distortion around titanium tandems. Since titanium doesn’t contain hydrogen atoms, tandems are dark in MRI images. In standard T2W MRI images, the uterus signal is typically very low due to the use of long echo time (TE). As a result, the contrast between the uterus and the tandem is pretty low, making the tandem delineation challenging. Using titanium applicators in MR-guided brachytherapy has been investigated in several studies [13-16]. Haack et al. reported that albeit very challenging, it was possible to assess titanium applicator geometry on T1 weighted (T1W) and T2W MRI images [14]. Wills et al. showed the feasibility of reconstructing titanium applicator using images from 0.35T open MRI scanner [15].

Reducing metal artifacts has been discussed substantially in MRI field for a long time and several sequences have been developed to address this issue [17-20]. However, those techniques remain mostly in research studies. They have not been widely adopted in clinical applications, primarily because 1) they are not very robust, and 2) most existing clinical MRI scanners don’t have the capability to incorporate work-in-progress sequences. Therefore, establishing new methods to reduce metal artifacts by exploiting the available product sequences and optimizing imaging parameters can be more beneficial to clinical work.

For MR-guided HDR brachytherapy for cervical cancer, the most commonly used sequence is T2W MRI because it can provide good tumor-tissue contrast [21]. However, using long TE in T2W MRI not only makes it very susceptible to susceptibility gradients caused by metal implants, but also significantly reduces the uterus signal and thus the contrast between the uterus and the titanium tandem. As a result, tandem delineation becomes a challenging and time consuming process. GEC-ESTRO (The Groupe Européen de Curiethérapie - the European Society for Therapeutic Radiology and Oncology) recommended that in some situation it can be can an option to acquire CT images or additional MRI image set to assist tandem delineation and streamline the whole process. Since CT scans involves unwanted radiation, acquiring another MRI image set is more preferable. Among available MRI sequences, T1W MRI is one option. Using T1W MRI to assist tandem delineation has been discussed previously in several studies [14,16,22]. In this study, we focused our effort on another commonly available MRI sequence - proton density weighted (PDW) MRI, which might be used to assist tandem delineation but hadn’t received enough discussion yet.

Compared to T2W MRI, PDW MRI uses a very short TE and a very long repetition time (TR) [23] which makes it highly SNR (signal to noise ratio) efficient and also less sensitive to susceptibility effect. High SNR efficiency can enhance the contrast between the uterus and the titanium tandem, making tandem delineation easier. In addition, it is possible to trade SNR for thinner slice, which helps mitigate the signal loss around titanium tandems. PDW MRI is available on almost all commercial MRI scanners, just like T2W MRI. Therefore, improved uterus-tandem contrast and reduced tandem delineation effort can be achieved on any existing MRI scanner.

## Methods

To show the effectiveness of using PDW MRI in assisting the delineation of the “tandem and ovoid” applicator, we retrospectively reviewed MRI images from cervical cancer patients who were treated with MR-guided HDR brachytherapy in our clinic and had both T2W and PDW images acquired. This study was approved by the Human Research Protection Office and the Protocol Review and Monitoring Committee of the Washington University School of Medicine. We retrospectively reviewed MRI images from ten patients treated from 11/2010 to 01/2011. The average patient age was 52.8±10.4 years.

After the placement of the applicator, cervical cancer patients were taken to the MRI suite for simulation. All MRI images were acquired on a 1.5T MRI scanner (Intera, Philips Medical Systems, Best, Netherlands). The body coil was used to transmit the Radiofrequency excitation pulse and a 4-channel pelvic coil was used to receive the signal. The imaging protocol started with a 3-plane localizer, followed by a reference scan to determine the coil sensitivity. After that, fat-saturated T2W images in axial plane were acquired. This image set was used to guide the subsequent para-sagittal images so that the tandem was parallel to the acquired para-sagittal plane and could be seen clearly as a whole in para-sagittal slices. T2W and PDW images in the para-sagittal plane were acquired subsequently. The Turbo spin echo (TSE) imaging technique was used in both sequences. Imaging parameters for T2W MRI are listed below. Slice thickness was 5 mm, gap between adjacent slices was 0 mm, in-plane resolution was 1 mm×1 mm and TE was 90 ms. The field of view (FOV) and the number of slices were determined according to patient’s anatomy. In PDW MRI, in-plane resolution was 1 mm×1 mm and TE was 5.5 ms. Since PDW MRI has high SNR efficiency, we were able to reduce slice thickness to 2.5 mm to decrease sequence’s sensitivity to susceptibility effect caused by metal implants but still maintain a good SNR for treatment planning. The number of slices and the in-plane FOV were kept the same as those used in T2W MRI. In slice-selected direction, PDW MRI had only half of the coverage compared to that provided by T2W MRI. The reduced coverage of PDW MRI in slice-select direction was justified as the main purpose of PDW MRI was to assist tandem delineation. The range of TR for both sequences was set between 3000 ms and 6000 ms, with the exact value determined by the scanner after optimization. After all scans were finished, images were sent to BrachyVision (Varian Medical Systems) for treatment planning and to the corresponding author’s workstation after de-identification for further image analysis.

During the entire treatment, each cervical cancer patient received 6 HDR brachytherapy procedures, or fractions. Since the improvement in uterus-tandem contrast is similar in all fractions, only images from the first fraction were used in this study. The comparison of T2W and PDW MRI were performed in both qualitative and quantitative methods.

Qualitatively, we compared T2W and PDW MRI images to determine which image sets had a better definition of the tandem. Signal profiles along a line which was perpendicular to the titanium tandem were obtained for both image sets. In addition, we segmented the tandem from surrounding tissues based on the histogram thresholding approach [24] and compared the segmented images obtained from T2W and PDW MRI images. In the histogram thresholding approach, one slice containing the majority portion of the tandem was selected from each image sets. A small region of interest (ROI) was placed around the tip of the tandem. Only two structures were included in the ROI: the tandem and the uterus. Histograms of the signal in the selected ROI were plotted for both image sets. Peaks in histograms corresponded to either the uterus or the tandem. Histograms were examined to see if peaks corresponding to the tandem and the uterus could be easily separated. A threshold was determined based on each histogram and was used to segment the corresponding MRI image. Voxels with signal intensity above the threshold (presumably the uterus) were set to 0. The rest of voxels (presumably the tandem) were set to 1. Structures other than the uterus and tandem are of little concerns in this segmentation process. The segmented images were visually inspected for the effectiveness of the segmentation.

Quantitatively, we compared the measured diameter of the tandem. The measurement was performed by an experienced medical physicist. On T2W images, the diameter of the tandem varies with the distance from the tip of the tandem. In addition, adjusting the windowing level also changes the measured diameter. To standardize the procedure of the measurement and reduce the corresponding bias as much as possible, the following two steps were performed during the measurement. First, the window level was determined based on the plastic ovoid because it has minimal geometric distortion in spin echo sequences. For each image, the window level was set so that the diameter of the ovoid was within 0.1 cm of the actual diameter. Second, the diameter of the tandem was measured at 1.5 cm from the tip of the tandem. The tip of the tandem was determined based on the PDW MRI image due to its better image quality. This measurement location was mapped to the co-registered T2W MRI image to ensure that the diameter was measured at the same location for both T2W and PDW images. The measured diameters of the tandem from MRI images were compared to the physical diameter, which was 0.345 cm determined by the caliper.

The contrast between the uterus and the tandem was also quantitatively determined for each patient and each image set. Again, the measurement was performed at the location which was 1.5 cm from the tip of the tandem. At this location, we drew a line which was perpendicular to the tandem. Along this line, two ROIs were placed. One was at the middle of the tandem and the other was 1.0 cm from the tandem in the uterus area. The size of the ROI within the uterus was 3 mm×3 mm, whereas the size of the ROI within the tandem was 1.5 mm×1.5 mm due to the small diameter of the tandem. The contrast can be determined by equation C = (S_u_ − S_t_)/S_u_, where S_u_ is the average signal in the uterus and S_t_ is the average signal in the tandem.

## Results

**Figure 1:**
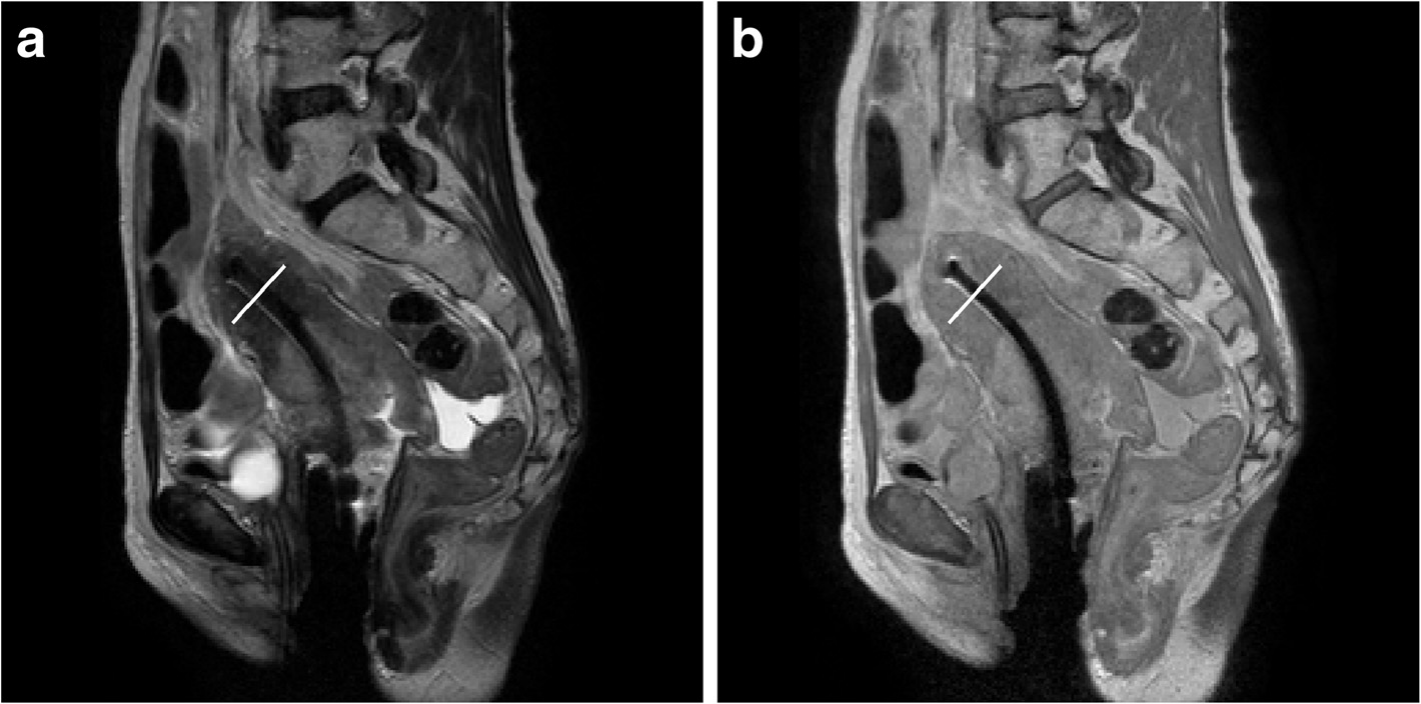
**Comparison of (a) T2W and (b) PDW MRI images for localizing tumors and the applicator.** White lines across the tandem on both images were added during the post processing to show the location of signal profiles plotted in Figure 2.

**Figure 2:**
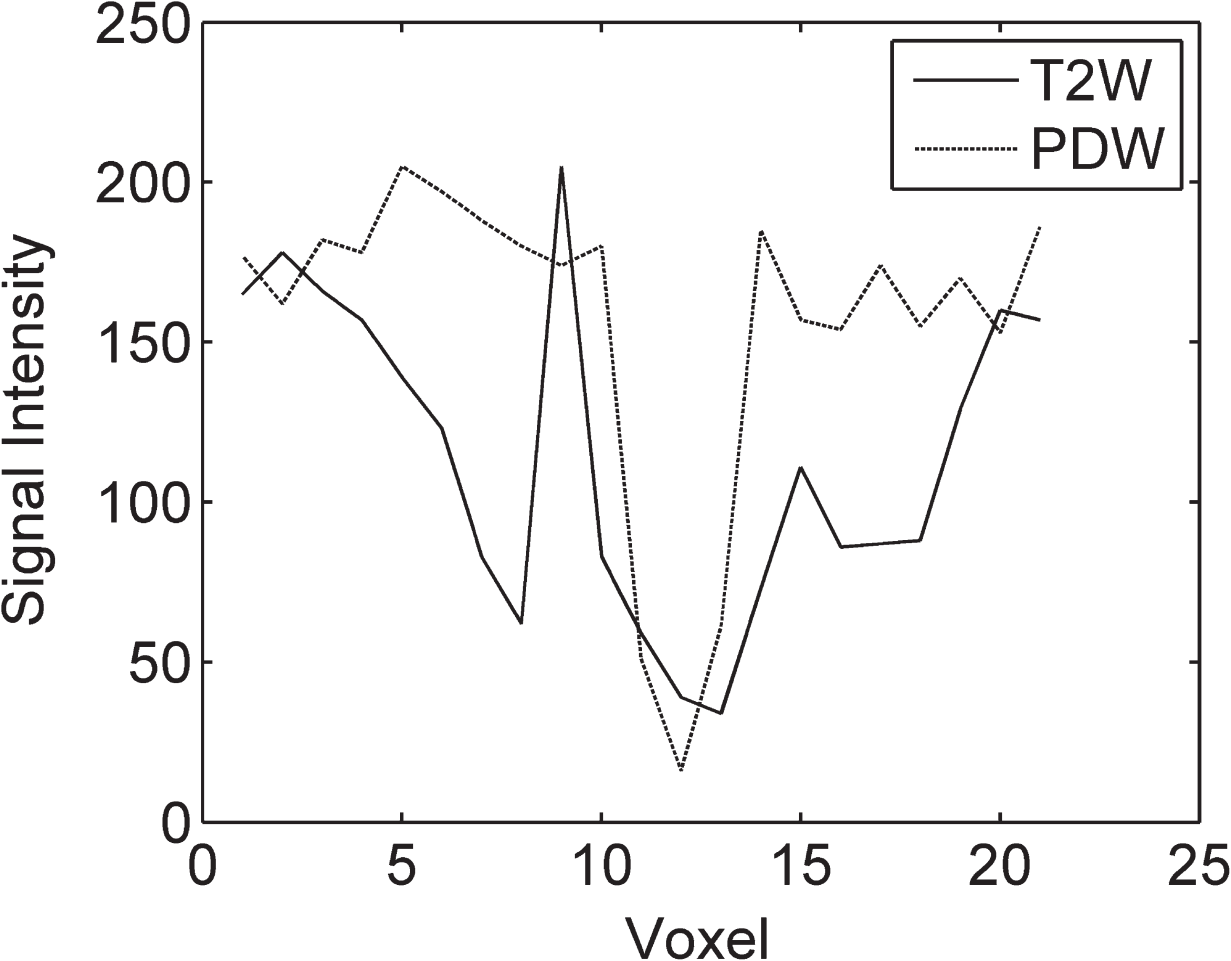
**Profiles across the tandem along the white lines shown in Figure**1**.** The PDW image show a uniform signal in the uterus, and a sharper and narrower tandem in the middle compared to the T2W image.

Figure 1 shows the comparison of T2W and PDW MRI images from a representative subject in localizing the tumor and the applicator. Qualitatively, the T2W image has a better tumor-tissue contrast. However, the low signal in the uterus reduces the contrast between the uterus and the tandem. In addition, the diameter of the tandem becomes wider towards the tip of the tandem. Therefore, T2W MRI can be a good candidate for tumor delineation but not a very good choice for defining the tandem. The PDW image, however, has more uniform and higher signal in the uterus even at 2.5 mm slice thickness, which makes it a lot easier to segment the tandem from the uterus. Figure 2 shows the comparison of profiles along the line perpendicular to the tandem. The location of the line is shown in Figure 1 around the top portion of the tandem. From Figure 2, it can be seen that the tandem looks narrower and sharper on PDW MRI than on T2W MRI. Table 1 lists the average signal in the uterus and the tandem. The uterus-tandem contrast was calculated for both image sets for all patients. The average uterus-tandem contrast from all patients was 0.42±0.24 for T2W MRI and 0.77±0.14 for PDW MRI. The improvement was statistically significant (p=0.0002).

**Table 1:** Measured uterus, tandem signal as well as the uterus-tandem contrast

**Patient**	**T2W**			**PDW**		
	**S**_**u**_	**S**_**t**_	**Contrast**	**S**_**u**_	**S**_**t**_	**Contrast**
1	134	66	0.51	217	31	0.86
2	126	89	0.29	190	28	0.85
3	95	88	0.07	189	55	0.71
4	133	49	0.63	189	40	0.79
5	248	128	0.48	191	60	0.69
6	148	87	0.41	213	38	0.82
7	108	42	0.61	225	14	0.94
8	85	82	0.04	177	100	0.44
9	172	37	0.78	210	41	0.80
10	96	64	0.33	123	28	0.77
AVE±STD			0.42±0.24			0.77±0.14
Paired student t-test			0.0002			

**Figure 3:**
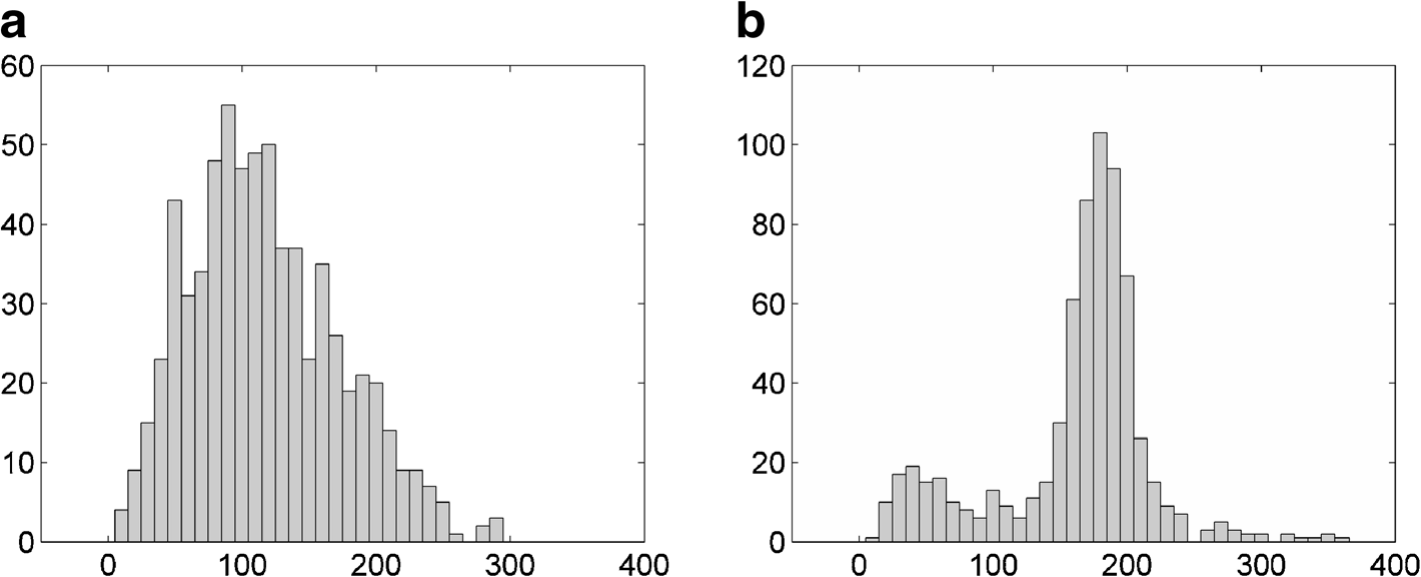
**Comparison of histograms of signal intensity between (a) T2W and (b) PDW MRI.** Histograms were obtained based on a small ROI placed around the tip of the tandem. Two peaks corresponding to the uterus and the tandem were overlapped in the T2W image but separable in the PDW image.

**Figure 4:**
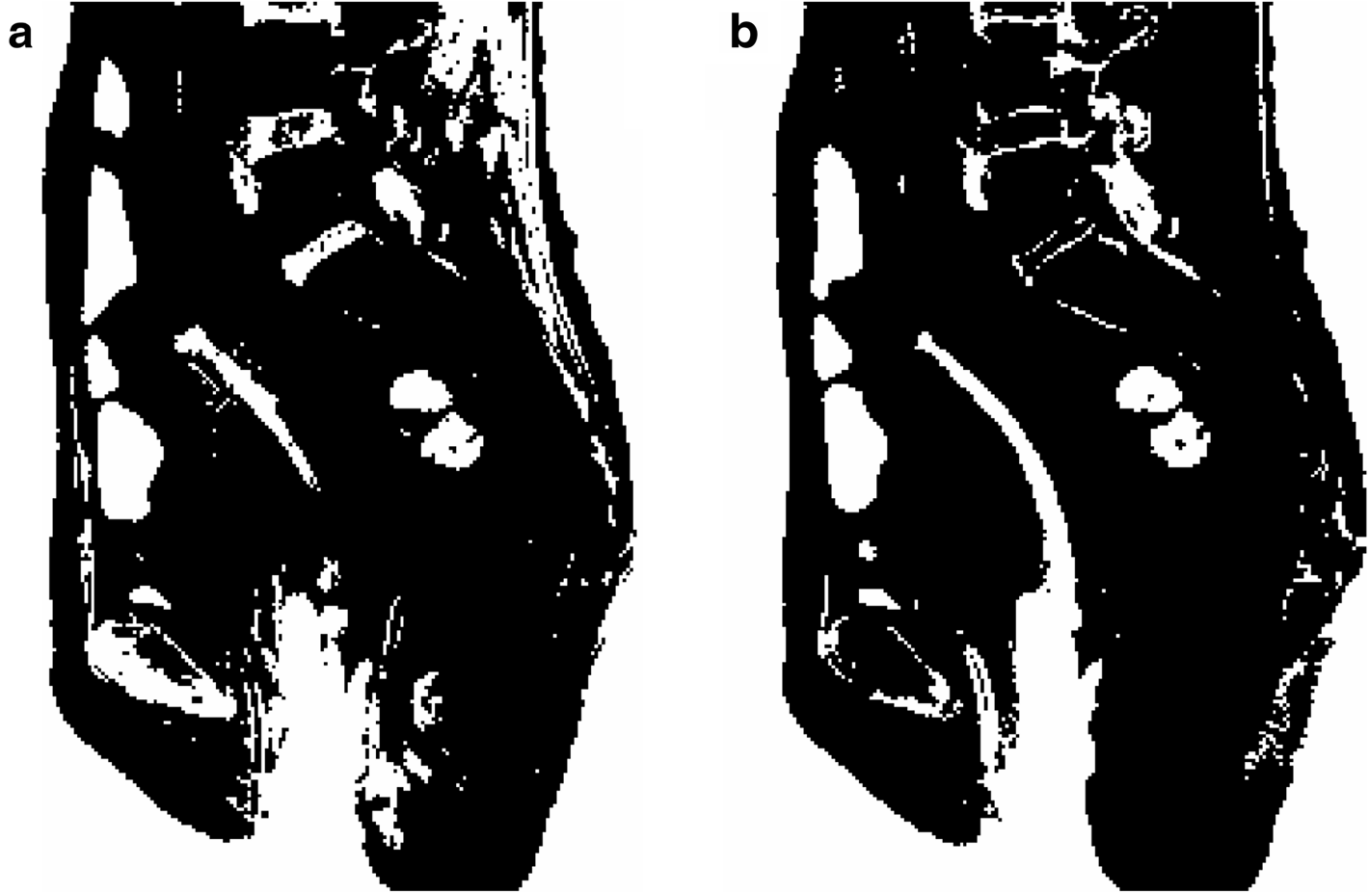
**Comparison of the segmented image using (a) T2W and (b) PDW images.** Image segmentation was based on the histogram thresholding technique. Thresholds were 70 and 90 for T2W and PDW images, respectively. The tandem looked nice and complete in the PDW image but quite fragmental in the T2W image.

To segment the tandem from the uterus using the histogram thresholding approach, a ROI was placed around the tip of the tandem. The selection of the ROI followed the steps described in the “Methods” section. Histograms of signal intensity in the ROI from T2W and PDW images are shown in Figure 3. Two peaks in the histograms corresponded to the uterus (the right peak) and the tandem (the left peak), respectively. For T2W MRI shown in Figure 3a, the two peaks overlapped with each other. Selecting a threshold to separate the two peaks was very challenge. In this case, a threshold of 70 was selected to segment the image. For PDW MRI shown in Figure 3b, the two peaks were clearly separable by using a threshold of 90. The segmented images using the thresholds determined by the histogram thresholding approach are shown in Figure 4. The tandem segmented from the PDW image looked more uniform and complete compared to that from the T2W image.

Table 2 lists the measured diameters of the tandem from the T2W and PDW images, differences between the measured diameter and the physical diameter (0.345 cm) of the tandem, the curvature of the tandem, and the physical diameter of the ovoid for each patient. The average measured diameters of the tandem from the T2W and PDW images were 0.54±0.15 cm and 0.45±0.11 cm, respectively. The average differences, or errors, between measured and physical diameters of the tandem were 0.20±0.15 cm for T2W MRI and 0.10±0.11cm for PDW MRI. The improvement by using PDW MRI was statistically significant (p=0.0003).

**Table 2:** Measured tandem diameters

**Patient**	**Tandem Curvature**	**Diameter of ovoid (cm)**	**Measured diameter (cm)**		**Difference from physical diameter (cm)**	
			**T2W**	**PDW**	**T2W**	**PDW**
1	30°	2.0	0.42	0.37	0.075	0.025
2	30°	2.0	0.32	0.29	−0.025	−0.055
3	30°	2.0	0.35	0.31	0.005	−0.035
4	45°	2.0	0.62	0.53	0.275	0.185
5	45°	1.6	0.49	0.39	0.145	0.045
6	45°	2.5	0.79	0.58	0.445	0.235
7	45°	2.5	0.61	0.53	0.265	0.185
8	45°	1.6	0.62	0.53	0.275	0.185
9	45°	2.5	0.68	0.55	0.335	0.205
10	45°	2.0	0.52	0.39	0.175	0.045
AVE±STD			0.54±0.15	0.45±0.11	0.20±0.15	0.10±0.11
Paired student t-test			0.0003		0.0003	

The curvature of the tandem, the physical diameter of the ovoid, the measured diameter of the tandem, as well as the difference between the measured diameter and the physical diameter (0.345 cm) from the T2W and PDW images for each patient are listed.

## Discussion

The feasibility of using PDW MRI to assist tandem delineation for cervical cancer patients treated with HDR brachytherapy was proved in this work. It was shown that PDW MRI had a better uterus-tandem contrast compared to T2W MRI. Qualitatively, the tandem had a better and more uniform shape on PDW MRI. Quantitatively, the difference between measured and physical diameters of the tandem was significantly reduced by using PDW MRI, confirming PDW MRI had reduced artifacts.

Although one of the standard methods to reduce geometric distortion and signal dropout is to increase the bandwidth and/or matrix size, it may not be a practical solution to improve image quality of T2W MRI and reduce effort in tandem delineation. In T2W MRI, the uterus signal is typically very low because of the use of long TE. The uterus-tandem contrast is barely enough for the delineation purpose. Increasing the bandwidth and/or matrix size, however, will significantly reduce the image SNR and thus the uterus-tandem contrast, making tandem delineation extremely difficult, if not impossible at all. PDW MRI uses a very short (5.5 ms vs. 90 ms). Therefore, the uterus signal is much higher in PDW MRI compared to T2W MRI, which not only increases the uterus-tandem contrast, but also allows us to use thinner slices to further reduce the geometric distortion and signal dropout around the titanium tandem. T1W MRI utilizes a TE which is comparable to PDW MRI. When T1W and PDW images are acquired using the same TE, bandwidth and spatial resolution, the two image datasets should exhibit similar amount of metal artifacts. On the other hand, T1W MRI utilizes a relatively shorter TR compared to PDW MRI, which makes it less SNR efficient. The long TR used in PDW MRI allows the magnetization to be fully relaxed before the next excitation. Therefore, more magnetization will be available at the beginning of each RF excitation, resulting in higher signal (or SNR) in PDW MRI. The SNR advantage of PDW MRI may be traded off for better spatial resolution, which will further reduce metal artifacts.

Image fusion between the T2W and PDW images was accomplished by using the coordinates stored in the DICOM header. This was warranted in this study because 1) the tabletop supporting patients did not move between the T2W and PDW MRI scans, all MRI images were acquired by changing the imaging hardware such as gradients; and 2) patients were still under medication for pain control which made them sleepy and less likely to move. None of the 10 patients included in this study showed significant movement between T2W and PDW MRI. If in future we do observe significant movement between scans, we can implement sophisticated fusion techniques based on patient’s anatomy to ensure the accuracy of the quantitative analysis.

The measured diameters from MRI images had a relatively large variance among patients. The largest measurement was 0.79 cm in one of the T2W images whereas the smallest measurement was 0.29 cm in one of the PDW images. The standard deviations in diameter measurement were 0.15 cm and 0.11 cm for T2W and PDW images, respectively. After examining Table 1, we noticed that there was a good correlation between the measured diameters from T2W images and PDW images. Specifically, if the measured diameter from the T2W image was large, the corresponding measured tandem diameter from the PDW image was also large and vice versa. In addition, we found that the measured diameter of the tandem correlated well with the curvature of the tandem. Patients using a tandem with bigger curvature tended to have a larger measured diameter. If patients were grouped by the curvature of the tandem, 3 patients had tandem curvature of 30° and 7 patients had tandem curvature of 45°. For the group with tandem curvature of 30°, the standard deviation of measured diameters reduced to 0.05 cm by using the T2W images and to 0.04 cm by using the PDW images. Similarly, for the group with tandem curvature of 45°, the standard deviation of measured diameters reduced to 0.10 cm by using the T2W images and to 0.08 cm by using the PDW images. We also noticed that some of the measured diameters were smaller than the physical diameter of the tandem. This may be explained by the relative location of the slices to the tandem. If the tandem is equally distributed into two adjacent para-sagittal slices along its axis, then each slice contains half of the tandem. Since the radius of the tandem is smaller than the slice thickness, for any voxels within the region of the tandem, it contains both tandem and the uterus. This partial volume effect may lower the contrast between the tandem and the uterus, making the measured diameter of the tandem smaller than its physical diameter. All these observations suggest that the measured diameter of the tandem is related to the curvature of the tandem, the location of slices, and the placement of the receiving coil and the applicator as well. Further investigation is needed and will be conducted in the future to fully understand the causes.

The focus of this retrospective study was to show the feasibility of using PDW MRI as an option to assist tandem delineation for cervical cancer patients treated with HDR brachytherapy. Other options to assist tandem delineation include acquiring additional x-ray images, CT images, T1W MRI or other MRI image-sets. Evaluating those options to find the best choice is a very interesting topic and of great clinical importance. However, it is out of the scope of this study. Such kind of study will be planned and performed in the future after being carefully designed to minimize the bias in the process of comparing images from different imaging modalities. Another interesting project is to compare dwell positions determined by MR images (T2W, T1W and PDW) and CT images. In our preliminary study on phantom [25], we found that mean differences between activated dwells on MRI vs. CT were approximately 0.1cm or less, 0.2 cm and 0.1 cm in RL (right-left), FH (foot-head), and AP (anterior-posterior) directions, respectively. Comparisons between T2W and PDW MRI showed about 0.1 cm difference in the FH direction and negligible differences in the other directions. Comparison of dwell positions using patient image datasets is currently under investigation.

## Conclusions

In this work, it was demonstrated that PDW MRI had a better uterus-tandem contrast compared to the standard T2W MRI and therefore could be used optionally to assist tandem delineation. The good uterus-tandem contrast provided by PDW MRI is complementary to the good tumor-tissue contrast offered by T2W MRI. By combing two images sets, it is possible to achieve both good tumor delineation and good tandem delineation simultaneously for cervical cancer patients treated with HDR brachytherapy.

## Abbreviations

HDR: High dose rate; CT: Computed tomography; MRI: Magnetic resonance imaging; T2W: T2 weighted; DWI: Diffusion weighted imaging; TE: Echo time; T1W: T1 weighted; GEC-ESTRO: The Groupe Européen de Curiethérapie - the European Society for Therapeutic Radiology and Oncology; PDW: Proton density weighted; TR: Repetition time; SNR: Signal to noise ratio; TSE: Turbo spin echo; FOV: Field of view; ROI: Region of interest.

## Competing interests

The authors declare that they have no competing interests.

## Authors’ contributions

YH designed the study, acquired and interpreted the data, and wrote the manuscript. JE analyzed and interpreted the data. SM, SR and HAG contributed to the interpretation of the data. JKS contributed to data acquisition. PWG contributed to the overall experimental design, data acquisition and interpretation. All authors have given final approval of the version to be published.
